# Editorial: Health and Children with Disabilities

**DOI:** 10.3389/fpubh.2017.00175

**Published:** 2017-07-17

**Authors:** Megan MacDonald, Samuel W. Logan

**Affiliations:** ^1^Oregon State University, Corvallis, OR, United States

**Keywords:** children, disabilities, health, young children, school-aged children

**Editorial on the Research Topic**

**Health and Children with Disabilities**

As indicated in the call for papers for the special topic on “Health and Children with Disabilities,” known health disparities exist among children with disabilities, including but not limited to motor, social, and communication delays. Addressing these known health disparities and ultimately improving the health and quality of life of children with disabilities is critical for moving toward community inclusiveness throughout educational and public health-related services. Given that child development is dynamic and occurs through the interaction of the individual, environment, and the task, it is important to understand key determinants affecting the health of children with disabilities at an individual and community level.

In this special topic, the editorial team welcomed contributions that employed various study types including single case studies, examination of key determinants of health, intervention, and relevant reviews, all targeted toward better understanding the health of children with disabilities. A majority of the contributing authors in this special topic are researchers within the field of *adapted physical activity*. This field embraces a broad perspective of inclusiveness and attitudes of acceptance. This perspective has been clearly articulated throughout this special topic and is thus an overarching theme throughout this e-book.

Articles within this e-book are focused on the health of children with disabilities and various frameworks have been used to articulate the dynamic interaction of the individual, environment, and the task as it relates to child health. For the purpose of this editorial, an age-related chronological order has been used to describe the content and related manuscripts in this special topic.

## Young Children

The health of young children with disabilities has been addressed within this e-book through various articles. In one study, the the co-occurrence of locomotion and peer interactions (i.e. social mobility) of young children with disabilities was compared to those without and examined across context (classroom, gymnasium, and playground) (Logan et al.). Differences in social mobility were found and how to overcome this gap between peers with and without disabilities was discussed. Allen-Meares et al. reviewed relevant information for early interventionists focused on autism spectrum disorder (ASD). In short, this review targeted key evidenced-based information to share with those at the forefront of working with families who have young children with ASD. In a small randomized control trial of young children in Head Start preschool programs, a motor skill-based intervention positively affected children’s motor skill development and self-regulation (Robinson et al.). In these articles, health is addressed holistically to include how children’s physical movement impacts aspects of social development for children with disabilities and the critical role of those on the front lines, like early interventionists, in providing up-to-date therapies to young children with disabilities.

## School-Aged Children

The health of school-aged children with disabilities was addressed through related reviews on mental and physical health of children as well as examination of community participation, including how to successfully transition from school into community life through self-empowerment. A review on developmental coordination disorder (DCD) highlighted specific characteristics of the disability, its likeliness to be diagnosed once a child enters school, and other related health issues common among children with DCD, including aspects of mental and physical health (Caçola). The good news is that studies have shown we can teach specific motor behaviors to children with DCD, which may positively impact aspects of health. MacDonald et al. examined the participation patterns of youth with Down syndrome (DS). This study highlighted how different activities had varied reach into the community and discussed how these findings might inform educational and community-based programs for school-aged children with DS. Sullivan studied students transitioning into community life, from high school, and examined how physical activity opportunities alongside peers of similar age empowered these students in their own life, and ultimately empowered students who received Special Education services to actively engage in the community. Taylor conducted a book review of A Teacher’s Guide to Adapted Physical Education: Including Students with Disabilities in Sports and Recreation, 4th Edition. This review is relevant for those teaching and providing health-related programs to school-aged students with disabilities and aligns with the broad perspective of inclusiveness and attitudes of acceptance that are central to the field of adapted physical activity/education.

Other important manuscripts addressed the health of children with disabilities, spanning young childhood into early adulthood. Dillon et al. conducted a systematic review and found that exercise is an evidence-based practice for individuals with ASD. Furthermore, collecting data and successfully measuring physical activity behaviors in children with disabilities can be difficult. Hauck et al. conducted a retrospective study and compared adherence strategies for wearing physical activity monitors in children with ASD. Strategies such as incentives, concealing techniques (e.g., monitors attached to clothing), and providing clear wear instructions can be used in practice to better capture objective physical activity behaviors in children with ASD. To that end, how we define physical activity behavior in children with disabilities differs across studies. Ross et al. examined current conceptual and methodological approaches evaluating physical activity participation of children with disabilities and provided recommendations as the field moves forward.

Finally, in the spirit of inclusiveness and attitudes of acceptance, Rimmer and Vanderbom articulate a *Call to Action*, for health researchers who work on obesity prevention programs. The *Call to Action* proposes collaboration with researchers in disability to adapt existing programs, so that they are inclusive to children with disabilities.

The known health disparities evident between children with and without disabilities are unacceptable. Together, researchers and practitioners can help eliminate this inequity. This special topic sheds light on some of these inequities but more importantly provides recommendations and suggests collaborations moving forward to overcome known health disparities for children with disabilities.

## Author Contributions

MM and SL worked together on this research topic and jointly reviewed manuscripts to write this editorial for the special topic Health and Children with Disabilities.

## Conflict of Interest Statement

The authors declare that the research was conducted in the absence of any commercial or financial relationships that could be construed as a potential conflict of interest.

